# Effects of Defatted Rice Bran Inclusion Level on Nutrient Digestibility and Growth Performance of Different Body Weight Pigs

**DOI:** 10.3390/ani11051374

**Published:** 2021-05-12

**Authors:** Bingbing Huang, Huangwei Shi, Li Wang, Lu Wang, Zhiqian Lyu, Qile Hu, Jianjun Zang, Defa Li, Changhua Lai

**Affiliations:** State Key Laboratory of Animal Nutrition, College of Animal Science and Technology, China Agricultural University, Beijing 100193, China; bbhuang217@163.com (B.H.); shw1161402802@163.com (H.S.); q15928806587@163.com (L.W.); wanglucau@163.com (L.W.); lzq2434390936@163.com (Z.L.); aaacheese@163.com (Q.H.); Zangjj@cau.edu.cn (J.Z.); Lidefa@cau.edu.cn (D.L.)

**Keywords:** body weight, defatted rice bran, digestibility, low-protein, performance, pigs

## Abstract

**Simple Summary:**

Feed grain, including corn and soybean meal, prices, which are the company’s primary raw materials, have fluctuated and escalated in recent years. Defatted rice bran, an abundant and underutilized agricultural coproduct of the paddy rice, can be used as a replacement. Additionally, nitrogen emitted as ammonia from swine manure has a negative effect on ambient air quality. This study evaluated the effects of defatted rice bran inclusion level in low-protein diets on growth performance and nutrient digestibility of different body weight pigs. Results showed that there is no difference for average daily gain for three weight stages, it meant that defatted rice bran could be used as a replacement for corns and soybean meal. Nutrient digestibility has significant difference. The study supported some theoretical foundation for the application of defatted rice bran.

**Abstract:**

This study was conducted to determine the effects of low-protein diet prepared with different levels of defatted rice bran (DFRB) and weight stages on growth performance and nutrient digestibility of growing–finishing pigs. The animal experiment included three stages. A total of 240 growing pigs with an initial body weight of 28.06 ± 8.56 kg for stage 1 were allocated to five diets including one control group and four DFRB diets supplemented with 2.5%, 5%, 7.5% and 10% DFRB, respectively. The 192 crossbred pigs with initial body weights of 55.03 ± 7.31 kg and 74.55 ± 9.10 kg were selected for stage 2 and stage 3, respectively. Pigs were allocated to four diets including one control group and three DFRB diets supplemented with 10%, 15% and 20% DFRB, respectively. The results showed that with the increase in DFEB intake, the gain: feed was linearly increased (*p* < 0.05), and the average daily feed intake tended to linearly decrease (*p* = 0.06) in stage 1. Except for the apparent total tract digestibility (ATTD) of acid detergent fiber (ADF) in stage 3, levels of DFRB had significant effects on the ATTD of gross energy (GE), dry matter (DM), ash, neutral detergent fiber (NDF) and ADF in three weight stages. In stage 1, with the increase in levels of DFRB, the ATTD of NDF and hemicellulose were firstly increased and then decreased (*p* < 0.01). In stage 2, with the increasing levels of DFRB, the ATTD of DM, ash and cellulose were firstly increased and then decreased (*p* < 0.01). In stage 3, the ATTD of GE, DM, ash, NDF and hemicellulose decreased linearly with the increase in levels of DFRB (*p* < 0.01). Collectively, DFRB could be used as a replacement for corns and soybean meal, and weight stage is important to consider when adjusting the additive proportion.

## 1. Introduction

China is a major pig-raising country in the world. Due to the structural contradiction between domestic supply and demand, because feed resources are gradually becoming in short supply, some crops are highly dependent on importation. In particular, the prices of imported commodities such as corn and soybean meal have soared over the years; unconventional feed ingredients have come into the spotlight, but how to make rational use of this resource will be crucial. Defatted rice bran (DFRB) is a by-product of rice that is nutritious and underutilized, which has a high protein content and a balanced amino acid composition [1,2]. It is a beneficial supplement to protein feed and may partly alleviate the shortage of protein feed. However, it is also rich in dietary fiber [3], which may limit its use by pigs. However, pigs with different body weights have different development of the gastrointestinal tract leading to varied digestion and fermentation abilities of dietary fiber, and this may also affect the digestibility of other nutrients and energy [4,5,6]. Therefore, it is necessary to study the effects of weight stage and inclusion levels of DFRB on growth performance and nutrient digestibility of pigs.

In addition, nitrogen (N) emitted as ammonia from swine manure has a negative effect on ambient air quality. Dietary manipulations may alter the pattern of nitrogen excretion, and subsequently reduce ammonia emissions [7]. Lowering dietary protein content, coupled with the use of crystalline amino acids to balance digestible amino acids will reduce urinary N excretion [8] and ammonia emissions [9]. Therefore, all the diets prepared in this study were low-protein diets.

Therefore, the objectives of this experiment were to (1) determine the suitable level of DFRB in different weight stages growing–finishing pigs; and (2) assess the effects of increasing levels of DFRB on performance and nutrient digestibility.

## 2. Materials and Methods

The protocol for this experiment was reviewed and approved by the China Agricultural University Laboratory Animal Welfare and Animal Experimental Ethical Inspection Committee (Beijing, China; No. AW90301202-1-1).

### 2.1. Animals and Housing

This study was conducted at the FengNing Swine Research Unit of China Agricultural University (Academician Workstation in Chengdejiuyun Agricultural and Livestock Co., Ltd., Beijing, China). The trial lasted for 97 days and was divided into three weight stages: 25–50 kg, 50–75 kg, and 75–100 kg.

Stage 1 included 240 growing pigs (Duroc × Landrace × Yorkshire; initial body weight 28.06 ± 8.56 kg) at the start of the experiment. Pigs were allocated randomly to 5 groups balancing for litter and gender. Stage 2 and 3 both included 192 growing pigs (Duroc × Landrace × Yorkshire; initial body weight 55.03 ± 7.31 kg and 74.55 ± 9.10 kg, respectively) at the start of the experiment. Pigs were allocated randomly to 4 groups balancing for litter and gender. Three weight stages pigs were housed in commercial flat-deck pens (8 pigs per pen, 6 pens per treatment). Room temperature was maintained at about 25 °C, and the humidity was maintained constant at about 65% to meet the environmental needs of the pigs. Each pen had one water nipple and one feed trough in the corner of the pen and the pens had rubber mats in approximately half of the pen area. Water and feed were provided ad libitum.

### 2.2. Experimental Diets

In stage 1, forty-eight pigs in each group received one of 5 experimental diets based on corn and soybean meal included the control (CON) diet, a 2.5% DFRB diet, a 5% DFRB diet, a 7.5% DFRB diet and a 10% DFRB diet. In stages 2 and 3, forty-eight pigs in each group received one of 4 experimental diets based on corn and soybean meal including the CON diet, a 10% DFRB diet, a 15% DFRB diet, and a 20% DFRB diet. The experimental diets meet the different pig weight stages’ nutrient requirements [10]. The amount of vitamin and mineral premix was kept constant in all diets. Acid-insoluble ash (AIA) was used as an indigestible marker. The chemical composition of diets is shown in Table 1 and nutrient levels of DFRB and diets are shown in Table 2.

### 2.3. Experimental Design and Sample Collection 

Growing–finishing pigs of each stage were randomly allotted to a completely randomized design with each stage of experimental diets. Each diet was replicated six times with eight pigs per replicate. Three weight stages lasted for 43, 24 and 30 d, respectively. Seven days of acclimation to diets and pens were allowed before the trial started. A sufficient adaptation was required before grab sampling of feces because high-fiber diets were used in the present experiment. The last 3 days of each stage were for feces collection.

The amount of feed provided for pigs was recorded and unconsumed feed was weighed daily for the determination of average daily feed intake (ADFI) [11]. Pigs were weighed individually at the beginning and end of each experiment stage. Average daily gain (ADG) was calculated as weight gain (final body weight—initial body weight) divided by the number of treatment days. Gain:feed was calculated as the ratio of ADG and ADFI [11]. A sample of each of the experimental diets was collected for chemical analysis. A fecal sample (300–500 g) was collected from each pen and pooled prior to drying. Feed and fecal samples were oven-dried at 65 °C for 72 h. All samples were ground to pass through a 40-mesh sieve for nutrient digestibility analysis.

### 2.4. Chemical Analyses

Samples of DFRB, diets and feces were analyzed for gross energy (GE) by automatic adiabatic oxygen bomb calorimetry (Parr 1281, Automatic Energy Analyzer, Moline, IL, USA). Acid insoluble ash (AIA) in the diets and feces was measured [12]. Samples of DFRB, diets and feces in this experiment were analyzed for dry matter (DM; method 930.15) [13], ash (method 942.05) [14], crude protein (CP; method 990.03) [13]. These samples were also analyzed for neutral detergent fiber (NDF) [15] and acid detergent fiber (ADF) [13] using fiber bags (model F57; Ankom Technology, Macedon, NY, USA) and a fiber analyzer (ANKOM^200^ Fiber Analyzer; Ankom Technology). Cellulose, hemicellulose, and lignin content of DFRB, diets and feces were measured [16]. Samples of DFRB and diets were analyzed for ether extract (EE) [14]. 

### 2.5. Calculation and Statistical Analyses

Organic matter (OM) was calculated as the difference between DM and ash. The apparent total tract digestibility of GE, DM, OM, ash, CP, NDF, ADF, cellulose, and hemicellulose were determined [17].

The UNIVARIATE procedure of SAS 9.2 (SAS Inst. Inc., Cary, NC, USA) was used to check the normality of residuals and equal variances. Outliers were identified as any value that deviated from the treatment mean by ± 3 times standard deviation. No outliers were observed in this experiment. Data on growth performance and nutrient digestibility were obtained using the GLM procedure of SAS (SAS Inst. Inc., Cary, NC, USA). The statistical model had diet as a fixed effect, pig as a random effect. The CONTRAST statement is used to determine linear and quadratic effect analysis. Treatment means were calculated using the LSMEANS statement and statistical significance was declared at *p* < 0.05.

## 3. Results

### 3.1. Chemical Analysis of Experimental Diets 

Chemical analysis of experimental diets is shown in Table 2. The GE of the diets for stage 1 was between 18.68 and 19.04 MJ/kg, the GE in diets for stage 2 was between 18.54 and 19.36 MJ/kg, the GE in diets for stage 3 was between 18.54 and 19.47 MJ/kg. The average CP content of all diets was 13.8% (Table 2). The analyzed concentration of fibrous fractions in diets was increased as DFRB increased in the diets. 

### 3.2. Effects of Defatted Rice Bran Inclusion Level on Nutrient Digestibility and Growth Performance of Different Body Weight Pigs 

The results are presented in Table 3, Table 4 and Table 5. The results showed that with the increase in DFRB intake, the feed conversion rate was linearly decreased (*p* < 0.05), and the ADFI tended to linearly decrease (*p* = 0.06) in stage 1. There were significant effects of level of DFRB on ATTD of GE, DM, ash, CP, NDF, and ADF in the different weight stages except for ATTD of ADF of stage 3. In stage 1, the ATTD of GE, DM, OM, CP, NDF, and ADF was lower in the basal diet than in diets containing DFRB. The ATTD of GE, DM and first ash increased and then decreased (Quadratic, *p* < 0.05) with increased DFRB intake, but NDF increased linearly (*p* < 0.01). In stage 2, the ATTD of nutrient digestibility first increased and then decreased (Quadratic, *p* < 0.05) with increased DFRB intake. In particular, 15% DFRB treatment had the highest nutrient digestibility compared to others. In stage 3, the ATTD of GE, DM, OM, ash, CP, NDF and hemicellulose decreased (Linearly, *p* < 0.01) with increased DFRB intake.

## 4. Discussion

The CP content of all diets is about 13~14%, which is in line with the expectations of low-protein diets. As shown in Table 3, Table 4 and Table 5, with the increase in DFRB inclusion level, the difference in ADFI and ADG of growing-finishing pigs at each body weight stage is not significant, which is consistent with previous studies [18,19]. It probably indicates that DFRB could be used as a replacement of corn and soybean meal under the inclusion level of this experiment. Warren et al. [18] found that the addition of 10% or 20% DFRB in the diet of growing pigs had no effect on growth performance. It is consistent with the test results. In stage 1, the gain:feed increased linearly and ADFI tended to decrease linearly as the inclusion level of DFRB increased from 2.5% to 10%. Casas et al. [20] found that the ADFI increased linearly, and the weight gain and feed ratio decreased linearly for finishing pigs as the inclusion level of DFRB increased from 0 to 30%. However, there are no effects on ADG for pigs at the three weight stages. It may be due to the DFRB maximum level of this experiment being lower. The ADFI and ADG in this experiment were lower than those reported by Casas et al. [20]. It may be because the DFRB that they used had higher GE and EE content, while the fiber content was lower. The higher the fiber content of DFRB, the more it will cause satiety and stop eating, which will affect the short-term feed intake. Oral sensory stimulation will affect the palatability of the feed [21,22]. Fiber fractions [23,24] and EE [25] both affect the digestibility of nutrients in pigs.

In stage 1, the ATTD of GE, DM, OM, CP, NDF, and ADF in the control group was lower than the DFRB diet. It shows that the DFRB diet can improve the digestibility of nutrients in pigs. With the increase in DFRB inclusion level, the ATTD of GE, DM, and ash first increased and then decreased, while the ATTD of NDF increased linearly. It may be that the digestibility of the soluble dietary fiber component contained in NDF is increasing, and the addition of DFRB to 10% exceeds the tolerance of pigs at this weight stage. In the second stage, the ATTD of nutrients increased first and then decreased with the increase in DFRB inclusion level. In particular, the 15% DFRB treatment group had the highest ATTD digestibility of nutrients. It may be due to the fact that the addition of DFRB at 10% promotes pig intestinal health [26] and then improves the digestibility of nutrients. The DFRB is rich in dietary fiber, and the content of total dietary fiber and soluble dietary fiber in DFRB is between 23.44~41.93% and 1.37~6.99%, respectively [1]. The soluble dietary fiber can be fermented by intestinal microorganisms and promote the colonization of beneficial intestinal flora [19]. When the inclusion level of DFRB increases to 20%, it may exceed the tolerance to DFRB fiber of pigs at this weight stage. Additionally, fiber increases the digesta passage rate and reduces the contact time between digesta and digestive enzymes [27]; finally, the digestibility of nutrients is reduced. In stage 2, with the increase in DFRB inclusion level, the ATTD of GE, DM, OM, Ash, NDF, CP and hemicellulose decreased linearly. It may be that the gastrointestinal tract of pigs has been developed relatively well when the body weight is over 75 kg, the prebiotic effect of fiber is not large, and the increase in fiber content affects the digestibility of other nutrients.

## 5. Conclusions

With the increase in the inclusion level of defatted rice bran, the average daily gain of pigs in the three weight stages is not affected, but the digestibility of nutrients is different. In terms of growth performance, the suitable levels of defatted rice bran for pigs at three weight stages in this experiment were 10%, 20% and 20%, respectively. The defatted rice bran could be used as a replacement for corns and soybean and become an important complement to protein feed.

## Figures and Tables

**Table 1:** Ingredient composition of the experimental diets (%, as-fed basis) ^1^.

Items	Weight Stage	25–50 kg					50–75 kg				75–100 kg			
	Diet	Control	2.5%	5%	7.5%	10%	Control	10%	15%	20%	Control	10%	15%	20%
Corn		80.20	77.30	74.70	71.88	69.50	80.63	70.08	64.73	59.63	81.27	70.43	65.13	59.93
Soybean meal		14.00	13.80	13.30	13.00	12.40	14.80	13.30	12.40	11.60	15.00	13.50	12.60	11.60
DFRB		-	2.50	5.00	7.50	10.00	-	10.00	15.00	20.00	-	10.00	15.00	20.00
Soybean oil		1.60	2.20	2.80	3.42	3.95	1.10	3.35	4.60	5.60	0.80	3.15	4.40	5.60
Dicalcium phosphate		1.40	1.30	1.20	1.20	1.00	1.30	0.80	0.70	0.50	0.80	0.50	0.40	0.15
Limestone		0.70	0.80	0.90	0.90	1.05	0.55	0.90	1.00	1.10	0.70	0.95	1.00	1.15
Salt		0.30	0.30	0.30	0.30	0.30	0.30	0.30	0.30	0.30	0.30	0.30	0.30	0.30
Vitamin–mineral premix ^2^		0.50	0.50	0.50	0.50	0.50	0.50	0.50	0.50	0.50	0.50	0.50	0.50	0.50
l-Lys HCl		0.68	0.68	0.68	0.68	0.68	0.50	0.50	0.50	0.50	0.40	0.40	0.40	0.40
dl-Met		0.19	0.19	0.19	0.19	0.19	0.09	0.09	0.09	0.09	0.05	0.09	0.09	0.09
l-Thr		0.25	0.25	0.25	0.25	0.25	0.15	0.15	0.15	0.15	0.15	0.15	0.15	0.25
l-Val		0.18	0.18	0.18	0.18	0.18	0.08	0.03	0.03	0.03	0.03	0.03	0.03	0.03

^1^ Diets with the same ingredient composition were formulated. ^2^ Vitamin–mineral premix for 25–50 kg pigs provided the following per kg of complete diet for growing pigs: vitamin A, 5512 IU; vitamin D_3_, 2200 IU; vitamin E, 30 IU; vitamin K_3_, 2.2 mg; vitamin B_12_, 27.6 μg; riboflavin, 4.0 mg; pantothenic acid, 14.0 mg; niacin, 30.0 mg; choline chloride, 400.0 mg; folacin, 0.7 mg; thiamine 1.5 mg; pyridoxine 3.0 mg; biotin, 44.0 µg; Mn (MnO), 40.0 mg; Fe (FeSO_4_·H_2_O), 75.0 mg; Zn (ZnO), 75.0 mg; Cu (CuSO_4_·5H_2_O), 100.0 mg; I (KI), 0.3 mg; Se (Na_2_SeO_3_), 0.3 mg. Vitamin–mineral premix for 50–75 kg and 75–100 kg pigs provided the following quantities per kilogram of the complete feed for growing pigs: vitamin A, 5512 IU; vitamin D_3_, 2200 IU; vitamin E, 64 IU; vitamin K_3_, 2.2 mg; vitamin B_12_, 27.6 µg; riboflavin, 5.5 mg; pantothenic acid, 13.8 mg; niacin, 30.3 mg; choline chloride, 551 mg; Mn (MnO), 40 mg; Fe (FeSO_4_·H_2_O), 100 mg; Zn (ZnO), 100 mg; Cu (CuSO_4_·5H_2_O), 100 mg; I (KI), 0.3 mg; Se (Na_2_SeO_3_), 0.3 mg.

**Table 2:** Nutrient and marker levels of the experimental diets and ingredients (%, DM basis).

Items ^1^	Weight Stage	25–50 kg					50–75 kg				75–100 kg				DFRB
	Diet	Control	2.5%	5%	7.5%	10%	Control	10%	15%	20%	Control	10%	15%	20%	
GE, MJ/kg		18.68	18.77	19.03	18.99	19.04	18.54	18.72	19.13	19.36	18.54	19.00	19.30	19.47	17.69
DM		87.81	87.72	87.72	88.00	87.95	87.45	88.01	88.11	88.46	87.07	87.81	87.98	88.37	89.22
OM		82.61	82.37	82.25	82.59	82.04	82.68	82.41	81.83	81.96	82.72	82.31	82.33	82.33	87.81
EE		2.55	2.61	3.55	3.53	4.68	2.52	3.63	4.69	5.90	1.47	4.69	6.33	7.73	0.50
CP		13.86	13.42	14.22	13.67	14.21	13.84	13.96	13.57	13.89	13.79	13.72	13.13	14.27	18.37
Ash		5.20	5.35	5.47	5.41	5.91	4.77	5.60	6.28	6.50	4.35	5.50	5.76	6.04	12.19
NDF		11.27	10.84	10.79	11.93	12.36	9.92	11.52	11.89	13.25	10.87	13.71	13.81	14.10	25.78
ADF		3.62	3.49	3.47	3.78	4.06	3.49	4.06	4.23	4.74	3.52	5.15	5.54	5.65	11.14
Lignin		0.00	0.00	0.00	0.15	0.00	0.00	0.20	0.13	0.23	0.04	0.43	0.31	0.57	2.30
Cellulose		3.38	3.24	3.31	3.44	3.73	3.34	3.65	3.98	4.27	3.36	4.41	4.68	4.82	7.99
Hemicellulose		7.65	7.35	7.31	8.15	8.31	6.43	7.46	7.66	8.52	7.35	8.56	8.27	8.46	13.06
AIA		0.44	0.46	0.40	0.40	0.45	0.48	0.60	0.52	0.66	0.44	0.51	0.72	0.79	-
Calculated values															-
SID Lysine		1.01	1.01	1.01	1.01	1.00	0.89	0.88	0.87	0.86	0.82	0.81	0.80	0.78	-
SID Methionine		0.39	0.39	0.39	0.39	0.39	0.30	0.29	0.29	0.29	0.26	0.29	0.29	0.29	-
SID Threonine		0.61	0.61	0.61	0.60	0.60	0.53	0.51	0.51	0.50	0.53	0.52	0.51	0.60	-
SID Tryptophan		0.28	0.28	0.27	0.27	0.27	0.28	0.27	0.27	0.26	0.29	0.27	0.27	0.26	-
SID Valine		0.68	0.68	0.68	0.68	0.67	0.59	0.54	0.53	0.53	0.55	0.54	0.54	0.53	-

^1^ GE, gross energy; DM, dry matter; OM, organic matter; EE, ether extract; CP, crude protein; NDF, neutral detergent fiber; ADF, acid detergent fiber; hemicellulose = NDF − ADF; AIA, acid insoluble ash.

**Table 3:** Effects of inclusion level of defatted rice bran on growth performance and apparent total tract digestibility in weight stage 1 ^1^.

Items	Weight Stage	25–50 kg					SEM	*p-*Value	
	Diet	Control	2.5%	5%	7.5%	10%		Linear	Quadratic
Growth performance									
Initial BW, kg		28.56	28.08	28.30	27.78	27.62	1.92	0.82	0.89
ADFI, g		1560	1727	1655	1587	1516	86.44	0.06	1.00
ADG, g		568	614	633	601	602	26.85	0.55	0.72
Gain:feed		0.36	0.36	0.38	0.38	0.40	0.01	<0.05	0.56
Final BW, kg		53.34	54.70	55.36	54.03	53.49	2.85	0.69	0.82
ATTD, %									
GE		83.80	85.96	86.58	87.10	86.02	0.40	0.66	<0.05
DM		84.93	86.50	86.84	87.29	85.93	0.35	0.38	<0.05
OM		86.55	88.26	88.51	88.95	88.23	0.36	0.81	0.16
Ash		55.32	55.36	58.04	58.35	54.96	0.97	0.84	<0.01
NDF		49.95	53.07	58.52	61.42	59.24	1.25	<0.01	<0.01
ADF		43.85	45.95	51.65	52.90	51.60	2.01	0.06	0.10
CP		74.46	79.73	79.84	81.02	80.46	1.16	0.39	0.70
Cellulose		56.74	52.73	58.43	58.02	57.38	3.60	0.08	0.07
Hemicellulose		59.62	56.45	61.78	65.38	62.98	3.24	<0.01	<0.01

^1^ Data are the least squares means of 6 observations for all diets. SEM, standard error of means; ADG, average daily gain; ADFI, average daily feed intake; ATTD, apparent total tract digestibility; GE, gross energy; DM, dry matter; OM, organic matter; CP, crude protein; NDF, neutral detergent fiber; ADF, acid detergent fiber; hemicellulose = NDF−ADF.

**Table 4:** Effects of inclusion level of defatted rice bran on growth performance and apparent total tract digestibility in weight stage 2 ^1^.

Items	Weight Stage	50–75 kg				SEM	*p-*Value	
	Diet	Control	10%	15%	20%		Linear	Quadratic
Growth performance								
Initial BW, kg		55.39	55.65	55.27	55.31	4.74	0.33	0.54
ADFI, g		2194	2435	2170	2199	113.91	0.12	0.79
ADG, g		830	865	798	805	35.22	0.10	0.90
Gain: feed		0.38	0.36	0.37	0.37	0.01	0.36	0.68
Final BW, kg		74.84	77.41	74.83	74.62	5.87	0.25	0.63
ATTD, %								
GE		84.55	82.13 ^c^	85.08	83.41	0.40	<0.01	<0.01
DM		85.72	82.12	84.83	82.59	0.36	0.20	<0.01
OM		87.45	84.78	87.05	79.61	1.21	<0.05	<0.05
Ash		51.39	37.34 ^c^	51.65	44.26	1.33	<0.01	<0.01
NDF		48.13	42.19 ^c^	53.24	48.72	1.80	<0.01	<0.01
ADF		43.91	33.15 ^c^	43.76	38.31	1.81	0.01	<0.01
CP		77.79	73.35	77.65	76.52	0.55	<0.01	<0.01
Cellulose		52.17	43.05 ^c^	54.42	49.16	1.10	<0.01	<0.01
Hemicellulose		50.43 ^c^	47.11 ^c^	58.47	54.51	1.23	<0.01	<0.01

^1^ Data are the least squares means of 6 observations for all diets. SEM, standard error of means; ADG, average daily gain; ADFI, average daily feed intake; ATTD, apparent total tract digestibility; GE, gross energy; DM, dry matter; OM, organic matter; CP, crude protein; NDF, neutral detergent fiber; ADF, acid detergent fiber; hemicellulose = NDF−ADF.

**Table 5:** Effects of inclusion level of defatted rice bran on growth performance and apparent total tract digestibility in weight stage 3 ^1^.

Items	Weight Stage	75–100 Kg				SEM	*p-*Value	
	Diet	Control	10%	15%	20%		Linear	Quadratic
Growth performance								
Initial BW, kg		74.73	74.55	74.76	76.6	5.58	0.33	0.46
ADFI, g		2682	2461	2472	2548	83.21	0.42	0.51
ADG, g		847	806	828	729	24.36	0.41	0.26
Gain: feed		0.32	0.33	0.33	0.29	0.01	0.86	0.28
Final BW, kg		101.11	99.39	100.64	99.85	5.74	0.33	0.40
ATTD, %								
GE		87.01	86.81	82.53	81.59	0.47	<0.01	<0.05
DM		88.07	87.2	82.55	80.79	0.44	<0.01	<0.05
OM		89.44	89.03	85.04	83.60	0.43	<0.01	<0.05
Ash		58.05	55.85	41.90	37.03 ^c^	1.03	<0.01	<0.01
NDF		56.78	62.44	49.29	46.70	2.68	<0.01	0.15
ADF		50.87	58.67	47.50	45.65	4.06	0.06	0.40
CP		82.82	82.48	75.13	74.49	0.66	<0.01	<0.01
Cellulose		58.63	63.43	53.41	53.93	3.61	0.11	0.29
Hemicellulose		59.61	64.70	50.49	47.40	2.10	<0.01	0.06

^1^ Data are the least squares means of 6 observations for all diets. SEM, standard error of means; ADG, average daily gain; ADFI, average daily feed intake; ATTD, apparent total tract digestibility; GE, gross energy; DM, dry matter; OM, organic matter; CP, crude protein; NDF, neutral detergent fiber; ADF, acid detergent fiber; hemicellulose = NDF−ADF.

## Data Availability

Data is contained within the article. The data presented in this study are available in Effects of Defatted Rice Bran Inclusion Level on Nutrient Digestibility and Growth Performance of Different Body Weight Pigs.
